# Crystal structure of (*E*)-2-(furan-2-yl­methyl­idene)-2,3,4,9-tetra­hydro-1*H*-carbazol-1-one

**DOI:** 10.1107/S2056989017017972

**Published:** 2018-01-01

**Authors:** A. Thiruvalluvar, M. Sridharan, K. J. Rajendra Prasad, M. Zeller

**Affiliations:** a Kunthavai Naacchiyaar Government Arts College for Women (Autonomous), Thanjavur 613 007, Tamilnadu, India; b Department of Chemistry, RV College of Engineering, Bangalore 560 059, Karnataka, India; c Department of Chemistry, Bharathiar University, Coimbatore 641 046, Tamilnadu, India; d Department of Chemistry, Purdue University, West Lafayette, IN 47907-2084, USA

**Keywords:** crystal structure, carbazol-1-one, furan, N–H⋯O hydrogen bonding, C–H⋯π inter­actions

## Abstract

The title compound, C_17_H_13_NO_2_, crystallizes with two conformationally very similar independent mol­ecules (*A* and *B*) in the asymmetric unit. In the crystal, the individual mol­ecules are linked by pairs of N—H⋯O hydrogen bonds forming *A*–*A* and *B*–*B* inversion dimers, with *R*_2_^2^(10) rings. They stack alternately up the *a*-axis direction and are linked by C—H⋯π inter­actions, forming sheets parallel to the *ab* plane.

## Abstract 2

The title compound crystallized with two conformationally very similar independent mol­ecules (A and B) in the asymmetric unit. In the crystal, the individual mol­ecules are linked by pairs of N—H⋯O hydrogen bonds, forming A–A and B–B inversion dimers, with 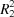
(10) rings.

## Chemical context   

Natural products comprising a carbazole skeleton linked to another heterocycle have received significant attention due to the promising anti­tumor properties of several of their naturally occurring representatives (Knölker & Reddy, 2002 ▸). Numerous total syntheses of these compounds have been reported that use a variety of structural modification methods for annelating heterocyclic systems to carbazole frameworks. This rapidly growing class of heteroaryl-condensed carbazoles has continued to attract attention because of their broad spectrum of useful biological activities that extend well beyond the anti­tumor properties of the naturally occurring carbazole derivatives that originally spiked the inter­est of researchers (Knölker & Reddy, 2002 ▸). Most heteroaryl carbazoles reported contain a heteroaryl moiety fused with a carbazole moiety; however, there are few reports where the heteroaryl unit is substituted with a carbazole unit (Sridharan *et al.*, 2008 ▸). We have reported the synthesis of 1-oxo-2-aryl­idene-2,3,4,9-tetra­hydro­carbazoles from potential precursors of the 2,3,4,9-tetra­hydro­carbazole-1-one type and these synthons were utilized to derive a diverse variety of heteroannelated carbazoles (Sridharan *et al.*, 2008 ▸; Sridharan & Rajendra Prasad, 2011 ▸; Archana *et al.*, 2010*a* ▸,*b* ▸; Thiruvalluvar *et al.*, 2013 ▸). Herein, we report on the crystal structure of one such compound, synthesized by the base-initialized reaction of 2,3,4,9-tetra­hydro­carbazol-1-one with furan-2-carbaldehyde.
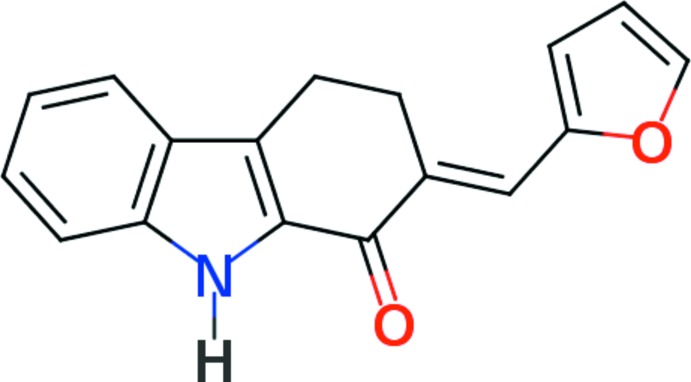


## Structural commentary   

**Figure 1:**
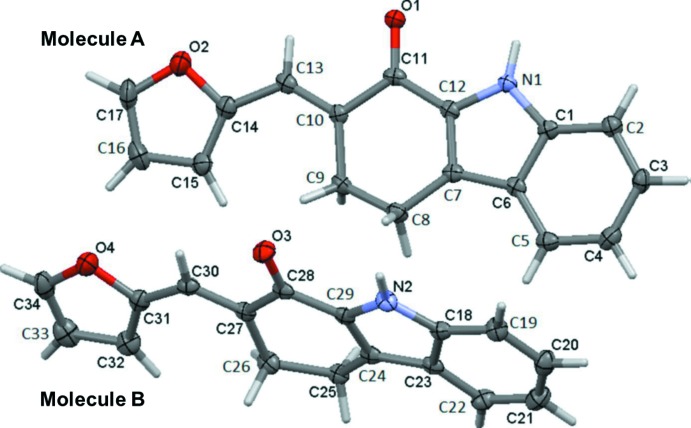
The mol­ecular structure of the two independent mol­ecules (*A* and *B*) of the title compound, with the atom labelling. Displacement ellipsoids are drawn at the 50% probability level.

**Figure 2:**
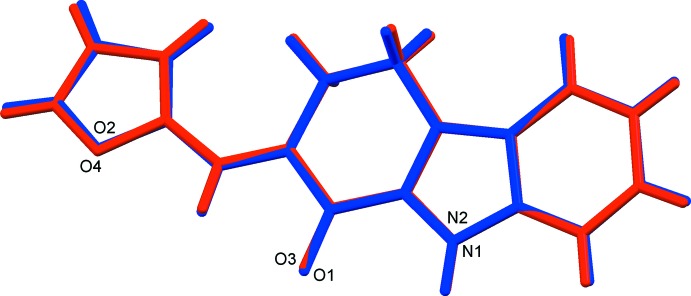
Mol­ecular overlay of inverted mol­ecule *B* (red) on mol­ecule *A* (blue).

The title compound, crystallizes with two independent mol­ecules (*A* and *B*) in the asymmetric unit (Fig. 1 ▸). The conformations of the two mol­ecules are similar, as can be seen in Fig. 2 ▸, which shows the mol­ecular overlay of mol­ecule *B* inverted on mol­ecule *A* (r.m.s. deviation = 0.082 Å). The cyclo­hexene rings of the tetra­hydro­carbazole moieties have half-chair conformations in both mol­ecules. The mean plane of the tetra­hydro­carbazole moiety (r.m.s. deviations are 0.087 and 0.072 Å for mol­ecules *A* and *B*, respectively) is inclined to the furan ring by 12.89 (14)° in mol­ecule *A*, and 12.09 (14)° in mol­ecule *B*.

## Supra­molecular features   

**Figure 3:**
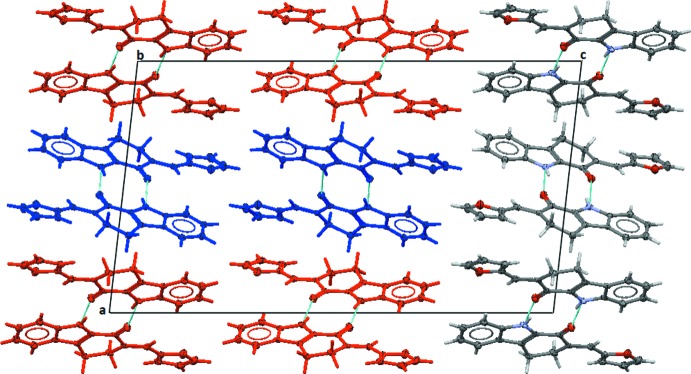
Crystal packing of the title compound, viewed along the *b* axis, showing the hydrogen bonded *A*–*A* and *B*–*B* inversion dimers, with 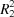
(10) ring motifs. The N—H⋯O hydrogen bonds are shown as dashed lines (see Table 1 ▸; mol­ecule *A* blue, mol­ecule *B* red).

**Figure 4:**
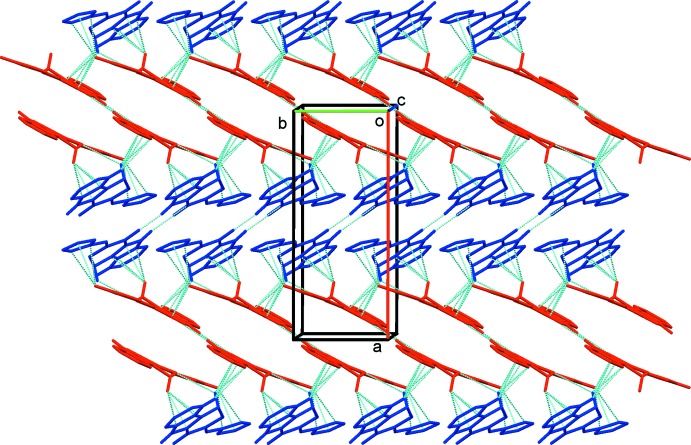
Crystal packing of the title compound, viewed along the *c* axis, showing the N—H⋯O hydrogen bonds and C—H⋯π inter­actions (blue dashed lines; see Table 1 ▸). Only the H atoms involved in these inter­actions have been included; *A* mol­ecules are blue and *B* mol­ecules are red.

In the crystal, the individual mol­ecules are linked by pairs of N—H⋯O hydrogen bonds forming *A*–*A* and *B*–*B* inversion dimers, with 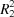
(10) ring motifs, which is the main motif that facilitates packing (Table 1 ▸ and Fig. 3 ▸). The individual dimers stack alternately along the *a*-axis direction, as shown in Fig. 3 ▸. The stacks are connected by C—H⋯π inter­actions, forming layers parallel to the *ab* plane (Fig. 4 ▸ and Table 1 ▸).

## Database survey   

A search in the Cambridge Structural Database (CSD, Version 5.38, update May 2017; Groom *et al.*, 2016 ▸) for the (*E*)-2-furyl­methyl­ene-2,3,4,9-tetra­hydro-1*H*-carbazol-1-one skel­eton gave four hits. These include (*E*)-2-[(furan-2-yl)methyl­idene]-7-methyl-2,3,4,9-tetra­hydro-1*H*-carbazol-1-one (CSD refcode: LESBAO; Thiruvalluvar *et al.*, 2013 ▸), 2-(2-furyl­methyl­ene)-6-methyl-2,3,4,9-tetra­hydro-1*H*-carbazol-1-one (OMABAG; Sridharan & Rajendra Prasad, 2011 ▸), (*E*)-2-(furan-2-yl­methyl­idene)-8-methyl-2,3,4,9-tetra­hydro-1*H*-carbazol-1-one (WACYAC; Archana *et al.*, 2010*a* ▸), and (*E*)-6-chloro-2-(furan-2-yl­methyl­idene)-2,3,4,9-tetra­hydro-1*H*-carbazol-1-one (WADDIQ; Archana *et al.*, 2010*b* ▸), which are closely related to the title compound. Half-chair conformations of the cyclo­hexene rings are observed in LESBAO, OMABAG and WACYAC, but a planar conformation is observed in the fourth structure, WADDIQ. The crystal packing in all four compounds, and the title compound, feature N—H⋯O hydrogen-bonded dimers with 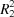
(10) ring motifs. LESBAO and OMABAG also exhibit C—H⋯O and C—H⋯π inter­actions, but such inter­actions are not present in WACYAC and WADDIQ.

## Synthesis and crystallization   

**Figure 5:**
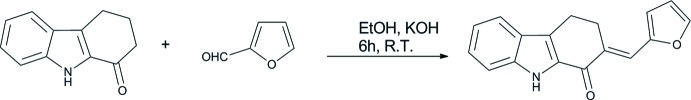
Synthesis of the title compound.

The synthesis of the title compound is illustrated in Fig. 5 ▸. An equimolar mixture of 2,3,4,9-tetra­hydro­carbazol-1-one (0.005 mol) and furan-2-carbaldehyde (0.005 mol) was treated with 25 ml of a 5% ethano­lic potassium hydroxide solution and stirred for 6 h at room temperature. The product precipitated as a yellow crystalline mass, which was filtered off and washed with 50% ethanol. A further crop of condensation product was obtained on neutralization with acetic acid and dilution with water. The product was recrystallized from ethanol to yield the title compound as yellow plate-like crystals (yield 1.17 g, 89%; m.p. 492–494 K).

## Refinement   

Crystal data, data collection and structure refinement details are summarized in Table 2 ▸. The NH H atoms, H1*A* and H2*B*, were located in a difference-Fourier map and freely refined. The remaining H atoms were placed in calculated positions, with C—H bond distances of 0.95 Å (aromatic H), and 0.99 Å (methyl­ene H), and refined as riding with *U*_iso_(H) = 1.2*U*_eq_(C). Reflections 002 and 100 were obstructed by the beam stop and omitted from the refinement.

## Supplementary Material

Crystal structure: contains datablock(s) I, Global. DOI: 10.1107/S2056989017017972/su5413sup1.cif

Structure factors: contains datablock(s) I. DOI: 10.1107/S2056989017017972/su5413Isup2.hkl

Supporting information file. DOI: 10.1107/S2056989017017972/su5413Isup3.cdx

CCDC reference: 1811751

Additional supporting information:  crystallographic information; 3D view; checkCIF report

## Figures and Tables

**Table 1:** Hydrogen-bond geometry (Å, °) *Cg*1, *Cg*2, *Cg*9, *Cg*10 are the centroids of rings O2/C14–C17, N1/C1/C6/C7/C12, N2/C18/C23/C24/C29 and C18–C23, respectively.

*D*—H⋯*A*	*D*—H	H⋯*A*	*D*⋯*A*	*D*—H⋯*A*
N1—H1*A*⋯O1^i^	0.93 (5)	1.90 (5)	2.792 (3)	160 (4)
N2—H2*B*⋯O3^ii^	0.89 (3)	1.91 (4)	2.788 (3)	168 (3)
C5—H5⋯*Cg*10	0.95	2.92	3.661 (3)	136
C8—H8*A*⋯*Cg*9	0.99	2.95	3.687 (3)	132
C25—H25*B*⋯*Cg*2^iii^	0.99	2.65	3.464 (3)	140
C33—H33⋯*Cg*1^iii^	0.95	2.92	3.564 (4)	126

Symmetry codes: (i) 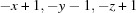; (ii) 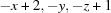; (iii) 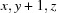.

**Table 2:** Experimental details

Crystal data	
Chemical formula	C_17_H_13_NO_2_
*M*_r_	263.28
Crystal system, space group	Monoclinic, *P*2_1_/*c*
Temperature (K)	100
*a*, *b*, *c* (Å)	15.353 (3), 6.3143 (13), 26.941 (6)
β (°)	96.446 (4)
*V* (Å^3^)	2595.3 (9)
*Z*	8
Radiation type	Mo *K*α
μ (mm^−1^)	0.09
Crystal size (mm)	0.43 × 0.14 × 0.06

Data collection	
Diffractometer	Bruker SMART APEX CCD
Absorption correction	Multi-scan (*SADABS*; Bruker, 2003 ▸)
*T*_min_, *T*_max_	0.707, 0.995
No. of measured, independent and observed [*I* > 2σ(*I*)] reflections	21411, 5293, 3646
*R*_int_	0.089
(sin θ/λ)_max_ (Å^−1^)	0.625

Refinement	
*R*[*F* ^2^ > 2σ(*F* ^2^)], *wR*(*F* ^2^), *S*	0.080, 0.147, 1.11
No. of reflections	5293
No. of parameters	369
H-atom treatment	H atoms treated by a mixture of independent and constrained refinement
Δρ_max_, Δρ_min_ (e Å^−3^)	0.25, −0.30

Computer programs: *SMART* and *SAINT-Plus* (Bruker, 2003 ▸), *SHELXTL* (Sheldrick, 2008 ▸), *Mercury* (Macrae *et al.*, 2008 ▸), *SHELXL2017* (Sheldrick, 2015 ▸), *PLATON* (Spek, 2009 ▸) and *publCIF* (Westrip, 2010 ▸).

## supplementary crystallographic information

### Crystal data

**Table:**

C_17_H_13_NO_2_	*D*_x_ = 1.348 Mg m^−^^3^
*M**_r_* = 263.28	Melting point: 493 K
Monoclinic, *P*2_1_/*c*	Mo *K*α radiation, λ = 0.71073 Å
*a* = 15.353 (3) Å	Cell parameters from 2429 reflections
*b* = 6.3143 (13) Å	θ = 2.7–30.5°
*c* = 26.941 (6) Å	µ = 0.09 mm^−^^1^
β = 96.446 (4)°	*T* = 100 K
*V* = 2595.3 (9) Å^3^	Plate, yellow
*Z* = 8	0.43 × 0.14 × 0.06 mm
*F*(000) = 1104	

### Data collection

**Table:**

Bruker SMART APEX CCD diffractometer	5293 independent reflections
Radiation source: fine-focus sealed tube	3646 reflections with *I* > 2σ(*I*)
Graphite monochromator	*R*_int_ = 0.089
ω scans	θ_max_ = 26.4°, θ_min_ = 1.9°
Absorption correction: multi-scan (SADABS; Bruker, 2003)	*h* = −19→19
*T*_min_ = 0.707, *T*_max_ = 0.995	*k* = −7→7
21411 measured reflections	*l* = −33→33

### Refinement

**Table:**

Refinement on *F*^2^	Primary atom site location: structure-invariant direct methods
Least-squares matrix: full	Secondary atom site location: difference Fourier map
*R*[*F*^2^ > 2σ(*F*^2^)] = 0.080	Hydrogen site location: mixed
*wR*(*F*^2^) = 0.147	H atoms treated by a mixture of independent and constrained refinement
*S* = 1.11	*w* = 1/[σ^2^(*F*_o_^2^) + (0.0269*P*)^2^ + 3.5969*P*] where *P* = (*F*_o_^2^ + 2*F*_c_^2^)/3
5293 reflections	(Δ/σ)_max_ < 0.001
369 parameters	Δρ_max_ = 0.25 e Å^−^^3^
0 restraints	Δρ_min_ = −0.29 e Å^−^^3^

### Special details

**Table:**

Geometry. All esds (except the esd in the dihedral angle between two l.s. planes) are estimated using the full covariance matrix. The cell esds are taken into account individually in the estimation of esds in distances, angles and torsion angles; correlations between esds in cell parameters are only used when they are defined by crystal symmetry. An approximate (isotropic) treatment of cell esds is used for estimating esds involving l.s. planes.

### Fractional atomic coordinates and isotropic or equivalent isotropic displacement parameters (Å^2^)

**Table:**

	*x*	*y*	*z*	*U*_iso_*/*U*_eq_	
C1	0.61376 (19)	−0.2774 (5)	0.60261 (12)	0.0173 (7)	
C2	0.6086 (2)	−0.3856 (5)	0.64790 (12)	0.0217 (7)	
H2	0.578325	−0.516373	0.649057	0.026*	
C3	0.6495 (2)	−0.2924 (5)	0.69026 (12)	0.0227 (7)	
H3	0.647151	−0.360851	0.721474	0.027*	
C4	0.6952 (2)	−0.0974 (6)	0.68913 (13)	0.0252 (8)	
H4	0.722265	−0.038237	0.719436	0.030*	
C5	0.7008 (2)	0.0072 (5)	0.64487 (12)	0.0216 (7)	
H5	0.732284	0.136616	0.644239	0.026*	
C6	0.65914 (18)	−0.0813 (5)	0.60055 (12)	0.0167 (7)	
C7	0.65038 (19)	−0.0186 (5)	0.54922 (12)	0.0177 (7)	
C8	0.6917 (2)	0.1602 (5)	0.52424 (12)	0.0216 (7)	
H8A	0.754404	0.127074	0.522669	0.026*	
H8B	0.688716	0.289636	0.544707	0.026*	
C9	0.6481 (2)	0.2046 (5)	0.47127 (12)	0.0203 (7)	
H9A	0.604123	0.318043	0.473396	0.024*	
H9B	0.693385	0.260999	0.451421	0.024*	
C10	0.60272 (19)	0.0196 (5)	0.44242 (11)	0.0165 (7)	
C11	0.57529 (19)	−0.1702 (5)	0.46952 (12)	0.0173 (7)	
C12	0.60027 (18)	−0.1724 (5)	0.52269 (11)	0.0165 (7)	
C13	0.58102 (19)	0.0203 (5)	0.39250 (12)	0.0199 (7)	
H13	0.553651	−0.104646	0.378684	0.024*	
C14	0.5942 (2)	0.1873 (6)	0.35736 (12)	0.0225 (7)	
C15	0.6212 (2)	0.3920 (5)	0.35988 (13)	0.0246 (8)	
H15	0.640850	0.468177	0.389436	0.030*	
C16	0.6147 (2)	0.4717 (6)	0.31037 (14)	0.0326 (9)	
H16	0.629385	0.610371	0.300397	0.039*	
C17	0.5837 (2)	0.3117 (7)	0.28029 (13)	0.0355 (9)	
H17	0.572466	0.321015	0.244960	0.043*	
N1	0.57937 (16)	−0.3319 (4)	0.55481 (9)	0.0179 (6)	
H1A	0.539 (3)	−0.439 (7)	0.5461 (17)	0.070 (15)*	
O1	0.53202 (14)	−0.3178 (3)	0.44786 (8)	0.0215 (5)	
O2	0.57065 (15)	0.1343 (4)	0.30757 (8)	0.0303 (6)	
C18	0.93890 (19)	0.1893 (5)	0.61166 (12)	0.0189 (7)	
C19	0.9632 (2)	0.0632 (5)	0.65399 (12)	0.0237 (8)	
H19	0.994298	−0.065812	0.651684	0.028*	
C20	0.9400 (2)	0.1351 (6)	0.69894 (13)	0.0279 (8)	
H20	0.954511	0.052622	0.728186	0.033*	
C21	0.8948 (2)	0.3294 (6)	0.70257 (13)	0.0299 (8)	
H21	0.880532	0.375123	0.734269	0.036*	
C22	0.8713 (2)	0.4529 (5)	0.66135 (12)	0.0235 (8)	
H22	0.840757	0.582267	0.664425	0.028*	
C23	0.89304 (19)	0.3854 (5)	0.61422 (12)	0.0184 (7)	
C24	0.87868 (19)	0.4659 (5)	0.56452 (12)	0.0178 (7)	
C25	0.8270 (2)	0.6529 (5)	0.54360 (12)	0.0202 (7)	
H25A	0.843140	0.777687	0.564918	0.024*	
H25B	0.763928	0.624526	0.545017	0.024*	
C26	0.8415 (2)	0.7065 (5)	0.48933 (12)	0.0229 (7)	
H26A	0.786330	0.766923	0.472647	0.028*	
H26B	0.886640	0.818814	0.490275	0.028*	
C27	0.86940 (18)	0.5264 (5)	0.45686 (12)	0.0175 (7)	
C28	0.91238 (19)	0.3342 (5)	0.48089 (12)	0.0178 (7)	
C29	0.91589 (18)	0.3218 (5)	0.53430 (12)	0.0176 (7)	
C30	0.86481 (19)	0.5329 (5)	0.40647 (12)	0.0201 (7)	
H30	0.884312	0.409242	0.390938	0.024*	
C31	0.8343 (2)	0.7024 (5)	0.37323 (12)	0.0221 (7)	
C32	0.8067 (2)	0.9061 (6)	0.37738 (13)	0.0267 (8)	
H32	0.798959	0.978925	0.407429	0.032*	
C33	0.7916 (2)	0.9893 (6)	0.32760 (14)	0.0319 (9)	
H33	0.772148	1.128015	0.318257	0.038*	
C34	0.8102 (2)	0.8329 (6)	0.29687 (14)	0.0332 (9)	
H34	0.805718	0.844498	0.261516	0.040*	
N2	0.95203 (17)	0.1539 (4)	0.56271 (10)	0.0191 (6)	
H2B	0.985 (2)	0.049 (6)	0.5523 (12)	0.026 (10)*	
O3	0.94238 (15)	0.1899 (4)	0.45586 (8)	0.0242 (5)	
O4	0.83651 (15)	0.6547 (4)	0.32307 (8)	0.0306 (6)	

### Atomic displacement parameters (Å^2^)

**Table:**

	*U*^11^	*U*^22^	*U*^33^	*U*^12^	*U*^13^	*U*^23^
C1	0.0095 (14)	0.0212 (17)	0.0213 (17)	0.0009 (13)	0.0028 (12)	−0.0031 (13)
C2	0.0188 (16)	0.0236 (18)	0.0227 (18)	−0.0002 (14)	0.0015 (13)	0.0023 (14)
C3	0.0180 (16)	0.0317 (19)	0.0188 (17)	0.0003 (15)	0.0036 (13)	0.0042 (15)
C4	0.0224 (17)	0.0325 (19)	0.0203 (18)	0.0018 (15)	0.0009 (14)	−0.0043 (15)
C5	0.0170 (16)	0.0219 (17)	0.0264 (19)	−0.0015 (14)	0.0040 (13)	−0.0058 (15)
C6	0.0093 (14)	0.0178 (16)	0.0235 (18)	0.0013 (12)	0.0044 (12)	−0.0004 (13)
C7	0.0138 (15)	0.0172 (16)	0.0222 (17)	0.0018 (13)	0.0032 (12)	−0.0032 (14)
C8	0.0190 (16)	0.0215 (17)	0.0241 (18)	−0.0035 (14)	0.0015 (13)	0.0004 (14)
C9	0.0180 (16)	0.0180 (17)	0.0255 (18)	−0.0030 (14)	0.0048 (13)	0.0020 (14)
C10	0.0116 (14)	0.0191 (16)	0.0191 (17)	0.0012 (13)	0.0032 (12)	0.0012 (13)
C11	0.0111 (14)	0.0157 (16)	0.0250 (17)	0.0013 (13)	0.0017 (12)	−0.0030 (14)
C12	0.0114 (14)	0.0159 (16)	0.0227 (17)	0.0012 (13)	0.0047 (12)	−0.0004 (13)
C13	0.0145 (15)	0.0188 (16)	0.0272 (19)	0.0005 (13)	0.0063 (13)	−0.0013 (14)
C14	0.0152 (15)	0.0297 (19)	0.0228 (18)	−0.0001 (15)	0.0036 (13)	0.0008 (15)
C15	0.0231 (17)	0.0285 (19)	0.0235 (19)	−0.0021 (15)	0.0081 (14)	−0.0023 (15)
C16	0.032 (2)	0.032 (2)	0.035 (2)	−0.0026 (17)	0.0110 (16)	0.0117 (18)
C17	0.038 (2)	0.048 (2)	0.021 (2)	−0.002 (2)	0.0056 (16)	0.0135 (19)
N1	0.0154 (13)	0.0198 (15)	0.0184 (14)	−0.0022 (12)	0.0017 (11)	0.0009 (12)
O1	0.0247 (12)	0.0194 (12)	0.0201 (12)	−0.0049 (10)	0.0014 (9)	−0.0001 (10)
O2	0.0334 (14)	0.0365 (15)	0.0211 (13)	−0.0048 (12)	0.0029 (11)	0.0018 (11)
C18	0.0125 (15)	0.0212 (16)	0.0240 (17)	−0.0023 (13)	0.0066 (13)	−0.0070 (14)
C19	0.0209 (17)	0.0248 (18)	0.0259 (19)	0.0035 (14)	0.0049 (14)	0.0018 (15)
C20	0.0234 (18)	0.038 (2)	0.0220 (18)	−0.0006 (16)	0.0006 (14)	0.0027 (16)
C21	0.0273 (19)	0.038 (2)	0.0250 (19)	0.0020 (17)	0.0045 (15)	−0.0061 (17)
C22	0.0223 (17)	0.0233 (18)	0.0251 (19)	0.0042 (15)	0.0029 (14)	−0.0044 (15)
C23	0.0116 (15)	0.0178 (16)	0.0260 (18)	−0.0012 (13)	0.0032 (13)	−0.0016 (14)
C24	0.0115 (14)	0.0182 (17)	0.0239 (18)	−0.0014 (13)	0.0030 (12)	−0.0019 (14)
C25	0.0177 (16)	0.0188 (17)	0.0248 (18)	0.0011 (14)	0.0055 (13)	−0.0018 (14)
C26	0.0188 (16)	0.0216 (18)	0.0294 (19)	0.0032 (14)	0.0072 (14)	0.0001 (15)
C27	0.0099 (14)	0.0181 (16)	0.0251 (18)	−0.0007 (13)	0.0050 (12)	−0.0021 (14)
C28	0.0109 (14)	0.0172 (16)	0.0263 (18)	−0.0026 (13)	0.0062 (12)	−0.0033 (14)
C29	0.0108 (14)	0.0180 (16)	0.0242 (17)	−0.0037 (13)	0.0026 (12)	−0.0007 (14)
C30	0.0161 (16)	0.0196 (17)	0.0247 (18)	−0.0010 (13)	0.0021 (13)	−0.0029 (14)
C31	0.0143 (16)	0.0297 (19)	0.0223 (18)	−0.0027 (15)	0.0026 (13)	0.0000 (15)
C32	0.0182 (17)	0.031 (2)	0.031 (2)	0.0030 (15)	0.0055 (14)	−0.0020 (16)
C33	0.0191 (18)	0.034 (2)	0.042 (2)	0.0046 (16)	−0.0001 (15)	0.0110 (18)
C34	0.0293 (19)	0.042 (2)	0.026 (2)	−0.0085 (18)	−0.0057 (15)	0.0138 (18)
N2	0.0162 (14)	0.0170 (14)	0.0250 (15)	0.0043 (12)	0.0063 (11)	0.0013 (12)
O3	0.0294 (13)	0.0213 (12)	0.0227 (12)	0.0047 (11)	0.0060 (10)	−0.0036 (10)
O4	0.0350 (14)	0.0331 (14)	0.0228 (13)	−0.0051 (12)	−0.0011 (11)	0.0020 (11)

### Geometric parameters (Å, º)

**Table:**

C1—N1	1.379 (4)	C18—N2	1.374 (4)
C1—C2	1.408 (4)	C18—C19	1.406 (4)
C1—C6	1.425 (4)	C18—C23	1.430 (4)
C2—C3	1.372 (4)	C19—C20	1.377 (5)
C2—H2	0.9500	C19—H19	0.9500
C3—C4	1.419 (5)	C20—C21	1.418 (5)
C3—H3	0.9500	C20—H20	0.9500
C4—C5	1.375 (5)	C21—C22	1.372 (5)
C4—H4	0.9500	C21—H21	0.9500
C5—C6	1.406 (4)	C22—C23	1.414 (4)
C5—H5	0.9500	C22—H22	0.9500
C6—C7	1.430 (4)	C23—C24	1.426 (4)
C7—C12	1.387 (4)	C24—C29	1.386 (4)
C7—C8	1.492 (4)	C24—C25	1.497 (4)
C8—C9	1.533 (4)	C25—C26	1.541 (4)
C8—H8A	0.9900	C25—H25A	0.9900
C8—H8B	0.9900	C25—H25B	0.9900
C9—C10	1.527 (4)	C26—C27	1.525 (4)
C9—H9A	0.9900	C26—H26A	0.9900
C9—H9B	0.9900	C26—H26B	0.9900
C10—C13	1.349 (4)	C27—C30	1.352 (4)
C10—C11	1.489 (4)	C27—C28	1.494 (4)
C11—O1	1.250 (4)	C28—O3	1.252 (4)
C11—C12	1.441 (4)	C28—C29	1.436 (4)
C12—N1	1.389 (4)	C29—N2	1.386 (4)
C13—C14	1.446 (4)	C30—C31	1.440 (5)
C13—H13	0.9500	C30—H30	0.9500
C14—C15	1.357 (5)	C31—C32	1.363 (5)
C14—O2	1.391 (4)	C31—O4	1.389 (4)
C15—C16	1.419 (5)	C32—C33	1.435 (5)
C15—H15	0.9500	C32—H32	0.9500
C16—C17	1.348 (5)	C33—C34	1.340 (5)
C16—H16	0.9500	C33—H33	0.9500
C17—O2	1.367 (4)	C34—O4	1.365 (4)
C17—H17	0.9500	C34—H34	0.9500
N1—H1A	0.93 (5)	N2—H2B	0.89 (3)
			
N1—C1—C2	129.4 (3)	N2—C18—C19	129.2 (3)
N1—C1—C6	108.5 (3)	N2—C18—C23	108.2 (3)
C2—C1—C6	122.0 (3)	C19—C18—C23	122.6 (3)
C3—C2—C1	116.6 (3)	C20—C19—C18	117.1 (3)
C3—C2—H2	121.7	C20—C19—H19	121.5
C1—C2—H2	121.7	C18—C19—H19	121.5
C2—C3—C4	122.4 (3)	C19—C20—C21	121.5 (3)
C2—C3—H3	118.8	C19—C20—H20	119.2
C4—C3—H3	118.8	C21—C20—H20	119.2
C5—C4—C3	121.0 (3)	C22—C21—C20	121.5 (3)
C5—C4—H4	119.5	C22—C21—H21	119.3
C3—C4—H4	119.5	C20—C21—H21	119.3
C4—C5—C6	118.6 (3)	C21—C22—C23	119.2 (3)
C4—C5—H5	120.7	C21—C22—H22	120.4
C6—C5—H5	120.7	C23—C22—H22	120.4
C5—C6—C1	119.4 (3)	C22—C23—C24	135.1 (3)
C5—C6—C7	134.1 (3)	C22—C23—C18	118.1 (3)
C1—C6—C7	106.6 (3)	C24—C23—C18	106.8 (3)
C12—C7—C6	106.9 (3)	C29—C24—C23	106.7 (3)
C12—C7—C8	122.5 (3)	C29—C24—C25	122.2 (3)
C6—C7—C8	130.3 (3)	C23—C24—C25	130.9 (3)
C7—C8—C9	113.2 (3)	C24—C25—C26	113.8 (3)
C7—C8—H8A	108.9	C24—C25—H25A	108.8
C9—C8—H8A	108.9	C26—C25—H25A	108.8
C7—C8—H8B	108.9	C24—C25—H25B	108.8
C9—C8—H8B	108.9	C26—C25—H25B	108.8
H8A—C8—H8B	107.7	H25A—C25—H25B	107.7
C10—C9—C8	117.4 (3)	C27—C26—C25	117.4 (3)
C10—C9—H9A	107.9	C27—C26—H26A	107.9
C8—C9—H9A	107.9	C25—C26—H26A	107.9
C10—C9—H9B	107.9	C27—C26—H26B	107.9
C8—C9—H9B	107.9	C25—C26—H26B	107.9
H9A—C9—H9B	107.2	H26A—C26—H26B	107.2
C13—C10—C11	116.1 (3)	C30—C27—C28	115.5 (3)
C13—C10—C9	123.5 (3)	C30—C27—C26	124.6 (3)
C11—C10—C9	120.3 (3)	C28—C27—C26	119.7 (3)
O1—C11—C12	121.7 (3)	O3—C28—C29	121.7 (3)
O1—C11—C10	122.4 (3)	O3—C28—C27	122.0 (3)
C12—C11—C10	115.9 (3)	C29—C28—C27	116.4 (3)
C7—C12—N1	109.9 (3)	C24—C29—N2	110.1 (3)
C7—C12—C11	125.2 (3)	C24—C29—C28	125.5 (3)
N1—C12—C11	124.9 (3)	N2—C29—C28	124.3 (3)
C10—C13—C14	128.2 (3)	C27—C30—C31	128.6 (3)
C10—C13—H13	115.9	C27—C30—H30	115.7
C14—C13—H13	115.9	C31—C30—H30	115.7
C15—C14—O2	108.8 (3)	C32—C31—O4	109.1 (3)
C15—C14—C13	136.6 (3)	C32—C31—C30	137.2 (3)
O2—C14—C13	114.6 (3)	O4—C31—C30	113.7 (3)
C14—C15—C16	107.6 (3)	C31—C32—C33	106.7 (3)
C14—C15—H15	126.2	C31—C32—H32	126.6
C16—C15—H15	126.2	C33—C32—H32	126.6
C17—C16—C15	106.4 (3)	C34—C33—C32	106.5 (3)
C17—C16—H16	126.8	C34—C33—H33	126.7
C15—C16—H16	126.8	C32—C33—H33	126.7
C16—C17—O2	110.8 (3)	C33—C34—O4	111.1 (3)
C16—C17—H17	124.6	C33—C34—H34	124.5
O2—C17—H17	124.6	O4—C34—H34	124.5
C1—N1—C12	108.0 (3)	C18—N2—C29	108.2 (3)
C1—N1—H1A	126 (3)	C18—N2—H2B	125 (2)
C12—N1—H1A	124 (3)	C29—N2—H2B	127 (2)
C17—O2—C14	106.5 (3)	C34—O4—C31	106.6 (3)
			
N1—C1—C2—C3	−179.9 (3)	N2—C18—C19—C20	−178.6 (3)
C6—C1—C2—C3	0.1 (4)	C23—C18—C19—C20	0.8 (5)
C1—C2—C3—C4	−0.2 (5)	C18—C19—C20—C21	−1.0 (5)
C2—C3—C4—C5	−0.4 (5)	C19—C20—C21—C22	0.9 (5)
C3—C4—C5—C6	1.0 (5)	C20—C21—C22—C23	−0.4 (5)
C4—C5—C6—C1	−1.1 (4)	C21—C22—C23—C24	178.5 (3)
C4—C5—C6—C7	179.3 (3)	C21—C22—C23—C18	0.1 (5)
N1—C1—C6—C5	−179.5 (3)	N2—C18—C23—C22	179.2 (3)
C2—C1—C6—C5	0.5 (4)	C19—C18—C23—C22	−0.3 (4)
N1—C1—C6—C7	0.2 (3)	N2—C18—C23—C24	0.3 (3)
C2—C1—C6—C7	−179.7 (3)	C19—C18—C23—C24	−179.2 (3)
C5—C6—C7—C12	−179.4 (3)	C22—C23—C24—C29	−179.1 (3)
C1—C6—C7—C12	0.9 (3)	C18—C23—C24—C29	−0.6 (3)
C5—C6—C7—C8	6.7 (6)	C22—C23—C24—C25	−5.3 (6)
C1—C6—C7—C8	−172.9 (3)	C18—C23—C24—C25	173.3 (3)
C12—C7—C8—C9	21.2 (4)	C29—C24—C25—C26	−16.9 (4)
C6—C7—C8—C9	−165.8 (3)	C23—C24—C25—C26	170.0 (3)
C7—C8—C9—C10	−27.5 (4)	C24—C25—C26—C27	26.2 (4)
C8—C9—C10—C13	−163.0 (3)	C25—C26—C27—C30	163.6 (3)
C8—C9—C10—C11	20.5 (4)	C25—C26—C27—C28	−22.1 (4)
C13—C10—C11—O1	−2.4 (4)	C30—C27—C28—O3	0.3 (4)
C9—C10—C11—O1	174.3 (3)	C26—C27—C28—O3	−174.4 (3)
C13—C10—C11—C12	178.7 (3)	C30—C27—C28—C29	−178.4 (3)
C9—C10—C11—C12	−4.6 (4)	C26—C27—C28—C29	6.8 (4)
C6—C7—C12—N1	−1.7 (3)	C23—C24—C29—N2	0.6 (3)
C8—C7—C12—N1	172.7 (3)	C25—C24—C29—N2	−173.9 (3)
C6—C7—C12—C11	179.7 (3)	C23—C24—C29—C28	176.3 (3)
C8—C7—C12—C11	−5.9 (5)	C25—C24—C29—C28	1.8 (5)
O1—C11—C12—C7	177.9 (3)	O3—C28—C29—C24	−174.9 (3)
C10—C11—C12—C7	−3.2 (4)	C27—C28—C29—C24	3.9 (4)
O1—C11—C12—N1	−0.5 (5)	O3—C28—C29—N2	0.2 (5)
C10—C11—C12—N1	178.4 (3)	C27—C28—C29—N2	179.0 (3)
C11—C10—C13—C14	175.9 (3)	C28—C27—C30—C31	−174.4 (3)
C9—C10—C13—C14	−0.7 (5)	C26—C27—C30—C31	0.1 (5)
C10—C13—C14—C15	−7.8 (6)	C27—C30—C31—C32	5.3 (6)
C10—C13—C14—O2	175.4 (3)	C27—C30—C31—O4	−178.6 (3)
O2—C14—C15—C16	0.0 (4)	O4—C31—C32—C33	−0.4 (4)
C13—C14—C15—C16	−176.9 (4)	C30—C31—C32—C33	175.9 (4)
C14—C15—C16—C17	0.3 (4)	C31—C32—C33—C34	0.3 (4)
C15—C16—C17—O2	−0.5 (4)	C32—C33—C34—O4	−0.2 (4)
C2—C1—N1—C12	178.7 (3)	C19—C18—N2—C29	179.5 (3)
C6—C1—N1—C12	−1.3 (3)	C23—C18—N2—C29	0.0 (3)
C7—C12—N1—C1	1.9 (3)	C24—C29—N2—C18	−0.4 (3)
C11—C12—N1—C1	−179.5 (3)	C28—C29—N2—C18	−176.2 (3)
C16—C17—O2—C14	0.5 (4)	C33—C34—O4—C31	−0.1 (4)
C15—C14—O2—C17	−0.3 (4)	C32—C31—O4—C34	0.3 (3)
C13—C14—O2—C17	177.4 (3)	C30—C31—O4—C34	−177.0 (3)

### Hydrogen-bond geometry (Å, º)

Cg1, Cg2, Cg9, Cg10 are the centroids of rings O2/C14–C17, N1/C1/C6/C7/C12, N2/C18/C23/C24/C29 and C18–C23, respectively.

**Table:**

*D*—H···*A*	*D*—H	H···*A*	*D*···*A*	*D*—H···*A*
N1—H1*A*···O1^i^	0.93 (5)	1.90 (5)	2.792 (3)	160 (4)
N2—H2*B*···O3^ii^	0.89 (3)	1.91 (4)	2.788 (3)	168 (3)
C5—H5···*Cg*10	0.95	2.92	3.661 (3)	136
C8—H8*A*···*Cg*9	0.99	2.95	3.687 (3)	132
C25—H25*B*···*Cg*2^iii^	0.99	2.65	3.464 (3)	140
C33—H33···*Cg*1^iii^	0.95	2.92	3.564 (4)	126

Symmetry codes: (i) −*x*+1, −*y*−1, −*z*+1; (ii) −*x*+2, −*y*, −*z*+1; (iii) *x*, *y*+1, *z*.
